# Functional Properties of Microorganisms in Fermented Foods

**DOI:** 10.3389/fmicb.2016.00578

**Published:** 2016-04-26

**Authors:** Jyoti P. Tamang, Dong-Hwa Shin, Su-Jin Jung, Soo-Wan Chae

**Affiliations:** ^1^Department of Microbiology, School of Life Sciences, Sikkim UniversityGangtok, India; ^2^Shindonghwa Food Research InstituteJeonju, South Korea; ^3^Clinical Trial Center for Functional Foods, Chonbuk National University HospitalJeonju, South Korea; ^4^Division of Pharmacology, Chonbuk National University Medical SchoolJeonju, South Korea

**Keywords:** fermented foods, microorganisms, functional properties, health benefits, bioactive compounds

## Abstract

Fermented foods have unique functional properties imparting some health benefits to consumers due to presence of functional microorganisms, which possess probiotics properties, antimicrobial, antioxidant, peptide production, etc. Health benefits of some global fermented foods are synthesis of nutrients, prevention of cardiovascular disease, prevention of cancer, gastrointestinal disorders, allergic reactions, diabetes, among others. The present paper is aimed to review the information on some functional properties of the microorganisms associated with fermented foods and beverages, and their health-promoting benefits to consumers.

## Introduction

Existing scientific data show many fermented foods have both nutritive and non-nutritive components in foods, which have the potential to modulate specific target functions in the body relevant to well-being and health of the consumers. However, 90% of naturally fermented foods and alcoholic beverages in different countries and regions of the world are still at home production under traditional conditions. Naturally fermented foods and beverages contain both functional and non-functional microorganisms (Tamang et al., 2016). Functional microorganisms transform the chemical constituents of raw materials of plant/animal sources during food fermentation thereby enhancing the bio-availability of nutrients, enriching sensory quality of the food, imparting bio-preservative effects and improvement of food safety, degrading toxic components and anti-nutritive factors, producing antioxidant and antimicrobial compounds, stimulating the probiotic functions, and fortifying with some health-promoting bioactive compounds (Tamang et al., 2009, 2016; Farhad et al., 2010; Bourdichon et al., 2012; Thapa and Tamang, 2015). Among bacteria associated with fermented foods and alcoholic beverages, lactic acid bacteria (LAB) mostly species of *Enterococcus*, *Lactobacillus*, *Lactococcus*, *Leuconostoc*, *Pediococcus*, *Weissella*, etc. are widely present in many fermented foods and beverages (Axelsson et al., 2012; Holzapfel and Wood, 2014). Species of *Bacillus* are also present in legume-based fermented foods (Kubo et al., 2011; Tamang, 2015). Species of *Bifidobacterium, Brachybacterium, Brevibacterium*, and *Propionibacterium* are isolated from cheese, and species of *Arthrobacter* and *Hafnia* from fermented meat products (Bourdichon et al., 2012). Several genera with hundred of species of yeasts have been isolated from fermented foods, alcoholic beverages and non-food mixed amylolytic starters which mostly include *Candida, Debaryomyces, Geotrichum, Hansenula, Kluyveromyces, Pichia, Rhodotorula, Saccharomyces, Saccharomycopsis, Schizosaccharomyces, Torulopsis, Wickerhamomyces*, and *Zygosaccharomyces* (Tamang and Fleet, 2009; Lv et al., 2013). Species of *Actinomucor, Amylomyces, Aspergillus, Monascus, Mucor, Neurospora, Penicillium, Rhizopus*, and *Ustilago* are reported for many fermented foods, Asian non-food amylolytic starters, and alcoholic beverages (Chen et al., 2014).

Functional properties of microorganisms in fermented foods include probiotics properties (Hill et al., 2014), antimicrobial properties (Meira et al., 2012), antioxidant (Perna et al., 2013), peptide production (De Mejia and Dia, 2010), fibrinolytic activity (Kotb, 2012), poly-glutamic acid (Chettri and Tamang, 2014), degradation of antinutritive compounds (Babalola, 2014), etc. which may be important criteria for selection of starter culture(s) to be used in the manufacture of functional foods (Badis et al., 2004). Some genera and species of microorganisms are used as commercial starters in food fermentation (**Table 1**), and some of products are commercialized and marketed globally as functional foods, health foods, therapeutic foods and nutraceuticals foods (Bernardeau et al., 2006; Bourdichon et al., 2012; Thapa and Tamang, 2015). The present paper is aimed to review the information on some functional properties of the microorganisms associated with fermented foods and beverages, and their health-promoting benefits to consumers.

**Table 1:** Some functional microorganisms used as commercial starters in food fermentation (amended and compiled from references: Mogensen et al., 2002; Bernardeau et al., 2006; Bourdichon et al., 2012; Thapa and Tamang, 2015).

Group	Genera/species	Product/application(s)
Bacteria
	*Acetobacter aceti* subsp. *aceti*	Vinegar
	*A. pasteurianus* subsp. *pasteurianus*	Vinegar, cocoa
	*Bacilllus acidopulluluticus*	Pullulanases (food additive)
	*B. coagulans*	Cocoa; glucose isomerase (food additive), fermented soybeans
	*B. licheniformis*	Protease (food additive)
	*B. subtilis*	Fermented soybeans, protease, glycolipids, riboflavin-B_2_ (food additive)
	*Bifidobacterium animalis* subsp. *lactis, B. breve*	Fermented milks with probiotic properties; common in European fermented milks
	*Brachybacterium alimentarium*	Gruyère and Beaufort cheese
	*Brevibacterium flavum*	Malic acid, glutamic acid, lysine, monosodium glutamate (food additives)
	*Corynebacterium ammoniagenes*	Cheese ripening
	*Enterobacter aerogenes*	Bread fermentation
	*Enterococcus durans*	Cheese and sourdough fermentation
	*E. faecium*	Soybean, dairy, meat, vegetables
	*Klebsiella pneumoniae* subsp. *ozaenae*	*Tempe;* production of vitamin B_12_
	*Lactobacillus acetototolerans*	Ricotta cheese, vegetables
	*L. acidophilus*	Fermented milks, probiotics, vegetables
	*L. alimentarius*	Fermented sausages; ricotta; meat, fish
	*L. brevis*	Bread fermentation; wine; dairy
	*L. buchneri*	Malolactic fermentation in wine; sourdough
	*L. casei* subsp. *casei*	Dairy starter; cheese ripening; green table olives
	*L. delbruecki* subsp. *bulgaricus*	Yogurt and other fermented milks, mozarella
	*L. fermentum*	Fermented milks, sourdough, urease (food additive)
	*L. ghanensis*	Cocoa
	*L. helveticus*	Starter for cheese; cheese ripening, vegetables
	*L. hilgardii*	Malolactic fermentation of wine
	*L. kefiri*	Fermented milk (*kefir*), reduction of bitter taste in citrus juice
	*L. kimchii*	*Kimchi*
	*L. oeni*	Wine
	*L. paracasei* subsp. *paracasei*	Cheese fermentation, probiotic cheese, probiotics, wine, meat
	*L. pentosus*	Meat fermentation and biopreservation of meat; green table olives; dairy, fruits, wine
	*L. plantarum* subsp. *plantarum*	Fermentation of vegetables, malolactic fermentation, green table olives; dairy, meat
	*L. sakei* subsp. *sakei*	Fermentation of cheese and meat products; beverages
	*L. salivarious* subsp. *salivarius*	Cheese fermentation
	*L. sanfranciscensis*	Sourdough
	*L. versmoldensis*	Dry sausages
	*Lactococcus lactis* subsp. *lactis*	Dairy starter, Nisin (protective culture)
	*L. lactis, L. mesenteroides* subsp. *Cremoris, L. mesenteroides* subsp. *Dextranicum, L. mesenteroides* subsp. *mesenteroides*	Dairy starter
	*Oenococcus oeni*	Malolactic fermentation of wine
	*Pediococcus acidilactici*	Meat fermentation and biopreservation of meat; cheese starter
	*P. pentosaceus*	Meat fermentation and biopreservation of meat
	*Propionibacterium acidipropionici*	Meat fermentation and biopreservation of meat
	*P. arabinosum*	Cheese fermentation; probiotics
	*P. freudenreichii* subsp. *freudenreichii*	Cheese fermentation (Emmental cheese starter)
	*Streptococcus natalensis*	Natamycin (food additive)
	*Weisella ghanensis*	Cocoa
	*Zymomonas mobilis* subsp. *mobilis*	Beverages
Yeasts
	*Candida famata*	Fermentation of blue vein cheese and biopreservation of citrus; meat
	*C. guilliermondii*	Citric acid (food additive)
	*C. krusei*	*Kefir* fermentation; sourdough fermentation
	*Debaryomyces hansenii*	Ripening of smear cheeses; meat
	*Geotrichum candidum*	Ripening of soft and semisoft cheeses or fermented milks; meat
	*Kluyveromyces marxianus*	Cheese ripening; lactase (food additive)
	*S. bayanus*	*Kefir* fermentation; juice and wine fermentation
	*S. cerevisiae*	Beer, bread, invertase (food additive)
	*S. cerevisiae* subsp. *boulardii*	Used as probiotic culture
	*S. florentius*	*Kefir* fermentation
	*S. pastorianus*	Beer
	*S. sake*	*Sake* fermentation
	*S. unisporus*	*Kefir* fermentation
	*Schizosaccharomyces pombe*	Wine


	*Zygosaccharomyces rouxii*	Soy sauce


Filamentous moulds
	*Aspergillus flavus*	α-amylases (food additive)


	*A. niger*	Beverages; industrial production of citric acid; amyloglucosidases, pectinase, cellulase, glucose oxidase, protease (food additives)


	*A. oryzae, A. sojae*	Soy sauce, beverages; α-amylases, amyloglucosidase, lipase (food additives)


	*Penicillium camemberti*	White mold cheeses (camembert type)


	*P. notatum*	Glucose oxidases (food additive)


	*P. roqueforti*	Blue mold cheeses


	*Rhizopus oligosporus*	*Tempe* fermentation


	*R. oryzae*	Soy sauce, *koji*




### Probiotic Microorganisms

Probiotics are defined as live microorganisms that, when administered in adequate amounts, confer a health benefit on the host (Hill et al., 2014). Probiotic organisms used in foods must have the ability to resist gastric juices, exposure to bile, and be able to proliferate and colonize the digestive tract (Saad et al., 2013). The beneficial effects of probiotic foods on human health and nutrition are constantly increasing (de LeBlanc et al., 2007; Monteagudo-Mera et al., 2012), and probiotics are popularly using bio-ingredients in many functional fermented foods (Chávarri et al., 2010). The most commonly used probiotic bacteria belong to the heterogeneous group of LAB (*Lactobacillus, Enterococcus*) and to the genus *Bifidobacterium*, however, yeasts and other microbes have also been developed as potential probiotics during recent years (Ouwehand et al., 2002). Some popular commercial probiotic cultures which are available in global markets include *Bacillus coagulans* BC30 marketed by Ganeden Biotech, Inc., Cleveland, OH, USA; *Lactobacillus acidophilus* NCFM, *Lactobacillus rhamnosus* HN001 (DR20) and *Bifidobacterium lactis* HN019 (DR10) marketed by Danisco (Madison, WI, USA), *L. casei* strain Shirota and *B. breve* strain Yakult marketed by Yakult (Tokyo, Japan), *L. fermentum* VRI003 (PCC) marketed by Probiomics (Eveleigh, NSW, Australia), *L. rhamnosus* R0011 marketed by Institut Rosell (Montreal, QC, Canada), *Streptococcus oralis* KJ3 marketed by Oragenics, Inc. (Alachua, FL, USA), and *Saccharomyces cerevisiae* (boulardii) marketed by Biocodex (Creswell, OR, USA; US Probiotics Home, 2011).

Products containing probiotic bacteria generally include foods and supplements (Varankovich et al., 2015). Fermented milk products are the most traditional source of probiotic strains of lactobacilli (Bernardeau et al., 2006; Shah, 2015); however, commercial probiotic lactobacilli have also been added to meat products, snacks, fruit juice, etc. (Ranadheera et al., 2010). Probiotic properties of *Lactobacillus plantarum* isolated from *kimchi*, Korean fermented vegetable product, has been reported (Ji et al., 2013), and is also found to prevent the growth of *Helicobacter pylori* (Lim and Im, 2009). Probiotic strain *L. acidophilus* La-5 produces conjugated linoleic acid (CLA), an anti-carcinogenic agent (Macouzet et al., 2009). *Pediococcus pentosaceus* CIAL-86 isolated from wine shows anti-adhesion activity against *Escherichia coli* CIAL-153, indicating its probiotic potential in wine (García-Ruiz et al., 2014).

### Antimicrobial Properties

Many species of LAB isolated from fermented vegetable and milk products have antimicrobial activities due to production of antimicrobial compounds such as bacteriocin and nisin (Tamang et al., 2009; Khan et al., 2010; Gaggia et al., 2011; Jiang et al., 2012; Grosu-Tudor and Zamfir, 2013). Many strains of LAB isolated from *kimchi* produce antimicrobial compounds such as bacteriocin by *L. lactis* BH5 (Hur et al., 2000) and *L. citreum* GJ7 (Chang et al., 2008), and pediocin by *P. pentosaceus* (Shin et al., 2008). Species of LAB isolated from *kimchi* show strong antimicrobial activity against *Listeria monocytogenes, Staphylococcus aureus, E. coli*, and *Salmonella typhimurium* (Lee et al., 2009). *Weissella cibaria* isolated from fermented cabbage product shows antimicrobial activity against Gram-positive and Gram-negative pathogens (Patel et al., 2014). *Lactococcus lactis* isolated from *dahi*, Indian curd, produces nisin Z that inhibits *L. monocytogenes* and *S. aureus* (Mitra et al., 2010). Several LAB species isolated from Romanian traditional fermented fruits and vegetables have antimicrobial activity against *L. monocytogenes*, *E. coli, Salmonella*, and *Bacillus* (Grosu-Tudor and Zamfir, 2013). Microorganisms as protective cultures, e.g., bacteriocin producers, may have several advantages, as they can contribute to the flavor, texture and nutritional value of the product besides the production of bacteriocin (Gaggia et al., 2011).

### Antioxidant Activity

Antioxidant activities in fermented foods include 1,1-diphenyl-2-picryl hydrazyl (DPPH) radical scavenging activity, 2,2′-azino-bis (3-ethylbenzo-thiazoline-6-sulfonic acid; ABTS) radical scavenging activity, total phenol content (TPC) estimation, and reducing power assay (Liu and Pan, 2010; Abubakr et al., 2012). Many Asian fermented soybean foods have antioxidant properties, e.g., *natto*, *Bacillus*-fermented soybean food of Japan (Ping et al., 2012), *chungkokjang* and *jang*, fermented soybean foods of Korea (Shon et al., 2007; Shin and Jeong, 2015), *douchi*, a fermented soybean food of China (Wang et al., 2007a), *kinema, Bacillus*-fermented soybean food of India and Nepal (Moktan et al., 2008; Tamang, 2015), *bekang* and *tungrymbai, Bacillus*-fermented soybean foods of India (Chettri and Tamang, 2014), *thua nao, Bacillus*-fermented soybean food of Thailand (Dajanta et al., 2013), and *tempe* mold-fermented soybean food of Indonesia (Nurrahman et al., 2013). Antioxidant activities have also been observed in *kimchi* (Park et al., 2011) and yogurt (Sabeena et al., 2010).

### Peptide Production

Bioactive peptides are formed during food fermentation by proteolytic microorganisms (De Mejia and Dia, 2010). In fermented foods peptides have some functional properties such as immunomodulatory (Qian et al., 2011), antithrombic (Singh et al., 2014), and antihypertensive properties (Phelan and Kerins, 2011). Species of *Bacillus* are involved in enzymatic hydrolysis of protein producing peptides and amino acids, which claim to have health benefits (Nagai and Tamang, 2010). Inhibitory properties of Angiotensin converting enzyme (ACE) have been studied in various fermented milk products such as *kefir* (Quiros et al., 2005), *koumiss* (Chen et al., 2010), yogurt (Papadimitriou et al., 2007), fermented camel milk (Moslehishad et al., 2013), cheese (Meyer et al., 2009), and fermented fish products (Ichimura et al., 2003).

### Production of Enzymes by Microorganisms

Another important reason to ferment foods is to coax microorganisms into producing enzymes that also provide very useful services. During food fermentation microorganisms produce enzymes to break down complex compounds to simple bio-molecules for several biological activities such as proteinase, amylase, mannase, cellulase, and catalase in many Asian fermented soybean foods by *Bacillus* spp. (Tamang and Nikkuni, 1996; Chettri and Tamang, 2014). Common genera of mycelial fungi in fermented foods and beverages such as *Actinomucor, Amylomyces, Aspergillus*, *Monascus, Mucor, Neurospora*, and *Rhizopus* produce various carbohydrases such as α- amylase, amyloglucosidase, maltase, invertase, pectinase, ß-galactosidase, cellulase, hemi-cellulase; acid and alkaline proteases; and lipases (Nout and Aidoo, 2002). Taka-amylase A (TAA), an enzyme produced by *Aspergillus oryzae* in *koji* has many uses in industry (Suganuma et al., 2007). Dry, solid, cake-like mixed amylolytic starters used for alcohol production in the Himalayas have yeasts *Saccharomycopsis fibuligera, S. capsularis* and *Pichia burtonii* with high amylase activities (Tsuyoshi et al., 2005; Tamang et al., 2007).

*Bacillus subtilis* subsp. *natto* in *natto* produces nattokinase showing fibrinolytic activity (Mine et al., 2005; Kotb, 2012). Among bacteria isolated from fermented foods, *B. subtilis* and *B*. *amyloliquefaciens* (Chang et al., 2012; Zeng et al., 2013; Singh et al., 2014), *Vagococcus carniphilus*, *V. lutrae*, *Enterococcus faecalis*, *E. faecium*, *E. gallinarum*, and *P. acidilactici* (Singh et al., 2014), and *Virgibacillus halodenitrificans* SK1-3-7 isolated from fish sauce fermentation (Montriwong et al., 2012) produce fibrinolytic enzymes.

### Increase in Isoflavones and Saponin and Production of PGA

Isoflavones are daidzein, genistein and glycitein, each of which exists in four chemical forms viz., aglycones, β-glucoside, acetylglucoside, and malonylglucoside in soybeans (Kudou et al., 1991). Isoflavone glucosides are hydrolyzed into their corresponding aglycones during fermentation of some Asian fermented soybean foods such as *sufu* and *douchi* of China (Wang et al., 2007b; Yin et al., 2007), *miso* and *natto* of Japan (Chiou and Cheng, 2001), *chungkokjang* and *doenjan*g of Korea (Lee et al., 2007), *tempe* of Indonesia (Lu et al., 2009), and *thua nao* of Thailand (Dajanta et al., 2009). During *tempe* fermentation, isoflavone particularly Factor-II and aglycone contents are found to increase (Nakajima et al., 2005). Isoflavones in *doenjan*g increase the activation of an LDL-C receptor, which is beneficial to prevent vascular diseases (Kwak et al., 2012).

Soybean saponins, which are oleanane triterpenoid glycosides, are again of two types viz., Group A and DDMP (2,3-dihydro-2,5-dihydroxy-6-metyl-4*H*-pyran-4-one; Paucar-Menacho et al., 2010). DDMP and their derivatives, Groups B and E saponins show health promoting benefits such as prevention of hypercholesterolemia (Murata et al., 2006), suppression of colon cancer cell proliferation (Ellington et al., 2006), and anti-peroxidation of lipids (Ishii and Tanizawa, 2006). Saponin contents are increased in *natto*, which are generated by *Bacillus natto* (Yanagisawa and Sumi, 2005). *Kinema* has high content of Group B saponin, which may indicate its health-promoting benefits to consumers (Omizu et al., 2011).

Poly-glutamic acid (PGA) is not synthesized by ribosomal proteins (Oppermann-Sanio and Steinbüchel, 2002), but is produced by some strains of *Bacillus* spp. in fermented soybean foods of Asia (Urushibata et al., 2002; Meerak et al., 2007; Nishito et al., 2010; Chettri and Tamang, 2014). *B. subtilis* and *B. licheniformis* are widely used industrial producers of γ-PGA (Stanley and Lazazzera, 2005). It is safe eating the viscous materials of Asian fermented soybean foods since PGA is completely biodegradable and water-soluble and non-toxic to human (Yoon et al., 2000).

### Degradation of Anti-nutritive Compounds

Some microorganisms present in fermented foods may degrade anti-nutritive substances and thereby convert the substrates into consumable products (Nout, 1994; Tamang, 2015). Various steps employed during the processing of *gari* and *fufu*, fermented cassava products of Africa, such as peeling, washing, grating, fermentation, dewatering and roasting minimizes the residual cyanide contents of the product (Babalola, 2014). Bitter varieties of cassava tubers contain the cyanogenic glycoside linamarin and lotaustralin, which are detoxified by species of *Leuconostoc, Lactobacillus*, and *Streptococcus* during traditional method to *gari* and *fufu* productions to yield hydrocyanic acid (HCN) which has low boiling point and escapes from the dewatered pulp during toasting rendering the product safe for human consumption (Lambri et al., 2013; Babalola, 2014; Bamidele et al., 2015). In *tempe*, *Rhizopus oligosporus* eliminates the flatulence causing indigestible oligosaccharides such as stachyose and verbascose into the absorbable monosaccharides and disaccharides (Hesseltine, 1983; Sanchez, 2008). Degradation of anti-nutritive compounds by *B. subtilis* has been reported in *kinema* (Sarkar et al., 1997). Phytic acid is reduced during fermentation of *idli* (Reddy and Salunkhe, 1980) and *rabadi*, a fermented cereal food of India (Gupta et al., 1992).

## Health Benefits of Fermented Foods

Ethnic foods have in-built systems both as foods and medicine to meet up hungry and also curative (Shin and Jeong, 2015; Thapa and Tamang, 2015). The highest longevity observed among the people of Okinawa prefecture in Japan is mostly due to their traditional and cultural foods such as *natto, miso, tofu, shoyu*, fermented vegetables, cholesterol-free, low-fat, and high bioactive-compounded foods in addition to active physical activity, sound environment, happiness and other several factors (Willcox et al., 2004). Korean *kimchi* has been claimed to possess health-promoting benefits (Cheigh, 1999; Lee et al., 2011; Park et al., 2014; Han et al., 2015). *Kimchi* has also anti-aging effect (Kim et al., 2002). *Natto* has several health benefits such as high contents of nattokinase, isoflavones, saponins, vitamin K, unsaturated fatty acids, probiotics and immunomodulating activities mostly produced by *B. subtilis* (*natto*; Tsubura, 2012; Nagai, 2015). *Kinema* has also some health promoting benefits (Omizu et al., 2011; Tamang, 2015). Indian popular fermented milk *dahi* has anti-carcinogenic property (Arvind et al., 2010). Lactic acid produced in *kimchi* may prevent fat accumulation and to improve obesity-induced heart diseases (Park et al., 2008). Anti-obesity effects have been reported in *kimchi* (Kim et al., 2011; Park et al., 2012) and in *doenjang* (Kwak et al., 2012) based on clinical trials (Cha et al., 2012; Jung et al., 2014). Red wine has anti-aging property due to presence of melatonin that regulates the body clock (Corder et al., 2006; Walker, 2014).

Ethnic people have customary belief in medicinal values of some of their ethnic foods including fermented foods and beverages, however, clinical trials and validation of the health benefits claims of almost all naturally fermented foods and beverages of the world need to be studied. Some health benefits of fermented foods are listed in **Table 2**.

**Table 2:** Some bioactive compounds in fermented foods and their health benefits.

Bioactive compounds	Synthesized in fermented foods	Health benefits	Reference
Genistein	*Doenjang*	Facilitates the β-oxidation of fatty acid, reducing body weight	Kwak et al., 2012
Lipoteichoic acid from *L. rhamnosus* GG	Fermented milk	Oral photoprotective agent against UV-induced carcinogenesis	Weill et al., 2013
Isocyanate and sulphide indole-3-carbinol	*Kimchi*	Prevention of cancer, detoxification of heavy metals in liver, kidney, and small intestine	Kwak et al., 2014
Ornithine		Anti-obesity efficacy	Park et al., 2012
Vitamin A, Vitamin C, fibers		Suppression of cancer cells	Han et al., 2015
Capsaicin, Allicin		Prevention of cancer, suppression of *Helicobacter pylori*	Lim and Im, 2009
Chlorophyll		Helps in prevention of absorbing carcinogen	Ferruzzi and Blakeslee, 2007
*S*-adenosyl-L-methionine (SAM)		Treatment of depression	Lee and Lee, 2009
HDMPPA (an antioxidant)		Therapeutic application in human atherosclerosis	Kim et al., 2007
Nattokinase, antibiotics, Vitamin K	*Natto*	Antitumor, immunomodulating	Nagai, 2015
Vitamin C	Sauerkraut	Scurvy	Peñas et al., 2013
Glucosinolates		Activation of natural antioxidant enzymes	Martinez-Villaluenga et al., 2012
Antioxidant genestein, daidzein, tocopherol, superoxide dismutase	*Tempe*	Prevents oxidative stress causing non-communicable disease such as hyperlipidemia, diabetes, cancer (breast and colon), prevents the damage of pancreatic beta cell	Astuti, 2015
Phenolics- resveratrol	*Wine (red)*	Anti inflammatory	Jeong et al., 2010
Phenolics, succinic acid		Digestive aid	Jackson, 2008
Phenolics, resveratrol, flavonoids – quercitin, Vitamins C and E, mineral selenium		Prevent cardiovascular diseases, reduce incidence of heart attacks and mortality rate	Walker, 2014
Melatonin, resveratrol		Antioxidant and anti-aging property	Fernández-Mar et al., 2012
Resveratol		Anti-diabetic	Ramadori et al., 2009


### Synthesis of Nutrient

Enrichment of substrates with vitamins, essential amino acids, and bioactive compounds occur during food fermentation (Holzapfel et al., 1995; Steinkraus, 1996; Thapa and Tamang, 2015). In *tempe*, mold-fermented soybean food of Indonesia, contents of folic acid, niacin, riboflavin, nicotinamide and pyridoxine are found to be increased by *Rhizopus oligosporus* (Astuti, 2015), whereas vitamin B_12_ is synthesized by non-pathogenic strains of *Klebsiella pneumoniae* and *Citrobacter freundii* (Liem et al., 1977; Okada, 1989; Keuth and Bisping, 1994). Contents of thiamine, riboflavin and methionine in *idli*, a rice-legume based fermented food of India and Sri Lanka enhance during fermentation (Ghosh and Chattopadhyay, 2011). Similarly, vitamins B complex and C, lysine and tryptophane, and iron contents have been found to increase during fermentation of *pulque*, an alcoholic drink of Mexico made from cactus plant (Ramirez et al., 2004). Riboflavin and niacin contents are increased in many *Bacillus*-fermented Asian fermented foods (Sarkar et al., 1998; Kim and Hahm, 2002; Nagai, 2015). Riboflavin and folic acid were found to be synthesized in *kimchi* by *L. mesenteroides* and *L. sakei* (Jung et al., 2013). Yeasts *Saccharomyces cerevisiae*, *Candida tropicalis*, *Aureobasidium* sp., and *Pichia manschuria* isolated from *idli* and *jalebi*, fermented cereal foods of India and Pakistan produce vitamin B_12_ (Syal and Vohra, 2013). Free amino acids are increased in fermented soybean foods (Nikkuni et al., 1995; Sarkar and Tamang, 1995; Tamang and Nikkuni, 1998; Kiers et al., 2000; Dajanta et al., 2011).

### Prevention of Hypertension and Heart Disease

Antihypertensive properties of many fermented milk products have been validated using animal models and clinical trials (Seppo et al., 2002; Sipola et al., 2002). Consumption of fermented milks or probiotic bacteria (Agerholm-Larsen et al., 2000) and fermented soybean foods (Liu and Pan, 2010) lowers the risk of heart diseases. Fermented whole grain foods can lower the serum LDL-cholesterol values, hypertriacylglycerolaemia, hypertension, coronary heart disease, insulin resistance, and hyperhomocysteinaemia (Anderson, 2003). Consumption of some fermented foods reduces the cholesterol level in *tempe* (Hermosilla et al., 1993), fermented soybean foods (Lee, 2004), and *kefir* (Otes and Cagindi, 2003). *Calpis*, the Japanese fermented sour milk containing two peptides VPP and IPP has shown hypotensive effect (Nakamura et al., 1996). *L. helvetius* in fermented milk reduces elevated blood pressure (Aihara et al., 2005; Shah, 2015). *Monascus purpureus* in fermented red-rice of China locally called *angkak*, prohibits creation of cholesterol by blocking a key enzyme, HMG-CoA reductase due to presence of mevinolin citrinin (Pattanagul et al., 2008).

Drinking of fermented tea of China prevents heart disease (Mo et al., 2008). Some Asian fermented soybean foods have antihypertensive properties as observed in *natto* (Nagai, 2015) and *tempe* (Astuti, 2015). Isoflavone in *doenjan*g, mold-fermented soybean food of Korea, plays an important role in preventing cardiovascular diseases (Kwak et al., 2012; Shin et al., 2015). Fermented whole-grain intake appears to protect from development of heart disease and diabetes (Anderson, 2003). Moderate consumption of wine is healthier (Walker, 2014). Polyphenols in red wine probably are synergists of the tocopherol (Vitamin E) and ascorbic acid (Vitamin C), thus they inhibit lipid peroxidation (Feher et al., 2007). Regular consumption of the Korean fermented soybean foods by hypertensive and Type 2 diabetic patients results in favorable changes in cardiovascular risk factors (Jung et al., 2014) and reduction of hypocholesterolemic effect (Lim et al., 2014). ACEs inhibitory peptides derived from food proteins are used for treating hypertension (Jakubczyk et al., 2013). Fermented foods, which are rich in fibrinolytic enzymes, are useful for thrombolytic therapy to prevent rapidly emerging heart diseases (Mine et al., 2005; Singh et al., 2014).

### Prevention from Cancer

Some LAB-fermented foods have antimutagenic and anticarcinogeinc activities (Lee et al., 2004). *Kefir* is used for the treatment of cancer (Otes and Cagindi, 2003; Yanping et al., 2009). Sauerkraut, fermented vegetable of Germany, contains *s*-methylmethionine, which reduces tumourigenesis risk in the stomach (Kris-Etherton et al., 2002). Consumption of fermented milk products containing live cells of *L. acidophilus* decreases ß-glucuronidase, azoreductase, and nitroreductase (catalyze conversion of procarcinogens to carcinogens), probably removes procarcinogens, and activate the immune system of consumers (Goldin and Gorbach, 1984; Macouzet et al., 2009). Similarly, Indian *dahi* has anti-carcinogenic property (Mohania et al., 2013). Cancer preventive potential of *W. cibaria*, and *L. plantarum* has been reported in *kimchi* (Kwak et al., 2014). Consumption of yogurt can reduce bladder, colon and cervical cancer has been observed (Chandan and Kilara, 2013).

### Protection against Gastrointestinal Disorders

Lactic acid bacteria present in fermented foods may decrease number of incidence, duration and severity of some gastrointestinal disorders (Verna and Lucak, 2010). Administration of some strains of *Lactobacillus* improves the inflammatory bowel disease, paucities and ulcerative colitis (Orel and Trop, 2014). *L. rhamnosus* GG is effective in the treatment of acute diarrhea (Szajewska et al., 2007) and administration of *L. helveticus*-fermented milk in healthy older adults produced improvements in cognition function (Chung et al., 2014). Consumption of fermented milk products containing live bacteria has immunomodulation capacity (Granier et al., 2013), and cures diarrhea (Balamurugan et al., 2014). Korean *kimchi* is suitable for control of inflammatory bowel diseases (Lim et al., 2011).

### Anti-allergic Reactions

*Lactobacillus kefiranofaciens* M1 isolated from *kefir* grains has an anti-allergic effect (Hong et al., 2010). Digestion of caseins during maturation of fermented milk products has shown to facilitate loss of allergenic reactivity thus increases tolerance (Alessandri et al., 2012). *Chongkokjang* has anti-allergic effect such as dermis thickness, decreased ear thickness, auricular lymph node and infiltrating mast cells (Lee et al., 2014). *Lactobacillus* species isolated from *kimchi* are found to modulate Th1/Th2 balance by producing a large amount of IL-12 and IFN-γ with ability to alleviate atopic dermatitis and food allergy (Won et al., 2011). Fermented fish oil, which is rich with Omega-3 polyunsaturated fatty acids, can reduce sensitization of allergy (Han et al., 2012).

### Protection from Diabetes and Osteoporosis

Intake of high fiber foods may decrease the insulin requirements in diabetic persons (Meyer et al., 2000), and may increase the sensitivity to insulin for non-diabetic persons (Fukagawa et al., 1990; Anderson, 2003). Probiotic *dahi*-supplemented diet significantly delays the glucose intolerance, hyperglycemia, hyperinsulinemia, oxidative stress and dyslipidemia indicating a lower risk of diabetes (Yadav et al., 2007). Daily consumption of *chungkokjang* may increase the insulin resistivity thus controls diabetics (Shin et al., 2011; Tolhurst et al., 2012).

Vitamin K2 present in *natto* stimulates the formation of bone, which may help to prevent osteoporosis in older women in Japan (Yanagisawa and Sumi, 2005). Mineral such magnesium, calcium, phosphorus, potassium, and also protein present in yogurt may function together to promote formation of healthy bones (Chandan and Kilara, 2013).

### Alleviation of Lactose Malabsorption

Some people suffer from lactose malabsorption, a condition in which lactose, the principal carbohydrate of milk, is not completely digested into glucose and galactose due to lack of ß-D-galactosidase (Shah, 2015). *L. delbrueckii* subsp. *bulgaricus* and *S. thermophilus* used in production of yogurt contain substantial quantities of ß-D-galactosidase which improve the symptoms of lactose malabsorption in lactose intolerant people (Shah et al., 2013). Consumption of fresh yogurt (with live yogurt cultures) has been demonstrated better lactose digestion and absorption than with the consumption of a pasteurized product (Pedone et al., 2000). *Kefir* can minimize the symptoms of lactose intolerance by providing extra source of β-galactosidase (Hertzler and Clancy, 2003).

## Health Risk of Fermented Foods

One of the important health risks in fermented foods is presence of biogenic amines. Biogenic amines are low molecular weight organic compounds by microbial decarboxylation of their precursor amino acids or by transamination of aldehydes and ketones by amino acid transaminases (Zhai et al., 2012), which are are present in some fermented foods such as *sauerkrau*t, fish products, cheese, wine, beer, dry sausages, etc. (Halász et al., 1994; Suzzi and Gardini, 2003; Spano et al., 2010; Visciano et al., 2014). Enterobacteriaceae and enterococci are major biogenic amine producers in foods (Nout, 1994). Foods with high levels of biogenic amines could be considered as unhealthy (Latorre-Moratalla et al., 2010). High levels (>100 mg/kg) of histamine and tyramine can cause adverse effects to human health (Rauscher-Gabernig et al., 2009). Fermentation of cabbage with certain lactic starters such as *L. casei* subsp. *casei, L. plantarum* and *L. curvatus* could reduce the biogenic amine content of *sauerkraut* (Rabie et al., 2011). The ingestion of food containing small amounts of histamine has little effect in healthy individuals, but it can result in histamine intolerance in persons characterized by impairment of diamine oxidase activity, either due to genetic predisposition, gastrointestinal diseases, or medication with monoamine oxidase inhibitors (Maintz and Novak, 2007). A maximum limit of 100 mg/kg of histamine in food indicates a safe level for consumption (Halász et al., 1994).

## Conclusion

Some fermented foods and beverages have health benefits due to presence of functional microorganisms. Although, some fermented foods and beverages are marketed globally as health foods, functional foods, therapeutic foods, nutraceutical foods, bio-foods, however, due to urbanization, changes in life-style, and the shifting from traditional food habits to commercial fast foods, the production and consumption of traditional fermented foods is in decline mostly in Asia and Africa. Reliance on fewer providers of fermented foods is also leading to a decline in the biodiversity of microorganisms. We recommend that validation of health claims by clinical trials and animal models of some common fermented foods of the world may be studied in details, and also introduction of new fermented food products containing well-validated functional microorganism(s) may emerge in global food market.

## Author Contributions

JPT (70% – data collection, analysis, writing), D-HS (10% – data collection), S-JJ (10% – data collection) and S-WC (10% – data collection).

## Conflict of Interest Statement

The authors declare that the research was conducted in the absence of any commercial or financial relationships that could be construed as a potential conflict of interest.
